# Valorization of Organic Food Surpluses and Brewer’s Spent Grains into Organic Insect Protein for Replacing Soybean in Post-Weaning Piglets

**DOI:** 10.3390/insects17060584

**Published:** 2026-06-03

**Authors:** Hugo Luttenschlager, Joachim Carpentier, Yves Beckers, José Wavreille, Nicolas Deville, Christophe Blecker, Sabine Danthine, Giorgia Purcaro, Philippe Maesen, Sandrine Dufourny, Fréjus Tanguy Ablo Zinsou, Aurore Richel, Frédéric Francis, Sébastien Finet, Rudy Caparros Megido

**Affiliations:** 1 Functional and Evolutionary Entomology, Gembloux Agro-Bio Tech, University of Liège, 5030 Gembloux, Belgium; joachim.carpentier@uliege.be (J.C.); nicolas.dvl@outlook.com (N.D.); frederic.francis@uliege.be (F.F.); 2 Precision Livestock and Nutrition, Gembloux Agro-Bio Tech, University of Liège, 5030 Gembloux, Belgium; yves.beckers@uliege.be; 3 Walloon Agricultural Research Centre, 5030 Gembloux, Belgium; j.wavreille@cra.wallonie.be (J.W.); s.dufourny@cra.wallonie.be (S.D.); 4 Unit of Food Science and Formulation, Research Unit, Teaching and Research Center (UR TERRA), Gembloux Agro-Bio Tech, University of Liège, 5030 Gembloux, Belgium; christophe.blecker@uliege.be (C.B.); sabine.danthine@uliege.be (S.D.); 5 Analytical Chemistry Laboratory, Gembloux Agro-Bio Tech, University of Liège, 5030 Gembloux, Belgium; gpurcaro@uliege.be; 6 Gembloux Environmental Engineering and Analysis Office (BEAGx), Gembloux Agro-Bio Tech, University of Liège, 5030 Gembloux, Belgium; philippe.maesen@uliege.be; 7 Département de Production Animale, Faculté des Sciences Agronomiques, Université d’Abomey-Calavi, Cotonou P.O. Box 03-2819, Benin; frejusablo@gmail.com; 8 Laboratory of Biomass and Green Technologies, Gembloux Agro-Bio Tech, University of Liège, 5030 Gembloux, Belgium; a.richel@uliege.be; 9 Biowaste Upcycling, 1410 Waterloo, Belgium; sf@zerowasteconsult.onmicrosoft.com

**Keywords:** *Hermetia illucens*, amino acids, supercritical CO_2_, circular economy, feed

## Abstract

The conversion of agro-industrial co-products and unsold organic plant-based residues into black soldier fly (BSF; *Hermetia illucens* (L. 1758)) proteins was assessed for use in organic post-weaning piglet diets in Belgium. A total of 72 crossbred female piglets (Landrace × Pietrain) were enrolled in a 5-week feeding trial. Experimental diets consisted of a common energy core (81.2% of the feed) and a protein core (18.8%) composed of organic soybean meal, pea meal, and potato protein, partially replaced by defatted BSF meal at inclusion levels of 15%, 25%, and 35%. All diets were formulated to be isoenergetic and isonitrogenous, with standardized ileal digestibility values for lysine, methionine, threonine, and tryptophan held constant. Incorporating 15% defatted BSF meal can substitute conventional organic protein sources without compromising growth performance in post-weaning piglets. However, economic modelling based on a cumulative feed conversion ratio expressed on a dry matter (DM) basis showed that break-even prices for organic BSF meal remained well below the price of the control protein nucleus (€1039·t^−1^, excluding VAT), indicating that economic parity could not be achieved at typical market prices under the observed feed efficiency.

## Simple Summary

This study explores the use of black soldier fly (BSF; *Hermetia illucens*) meal produced from agro-industrial co-products and unsold organic plant residues as a protein source in organic diets for post-weaning piglets. Seventy-two piglets were fed diets in which conventional organic protein sources were partially replaced by defatted BSF meal at different inclusion levels. A 15% inclusion of BSF meal can replace traditional protein ingredients without negatively affecting growth performance. However, an economic assessment based on feed efficiency indicates that the cost of BSF meal remains too high to compete with conventional protein sources under current market conditions. These findings highlight the nutritional potential of BSF meal in organic pig production, while underlining the need for improved economic viability.

## 1. Introduction

Globally, soybean meal is the primary protein source used in monogastric animal nutrition, particularly in pigs and poultry [1,2]. World soybean production reached 427.15 million metric tons in the 2024–2025 season [3], primarily driven by the growing demand for animal feed. A major sustainability concern associated with soybean production is its contribution to deforestation in the Amazon rainforest [4,5]. This situation drives biodiversity loss and land use changes, releasing a significant share of soil organic carbon stock as CO_2_ in the atmosphere [6]. In addition, intensive soybean production places a double strain on groundwater and surface water through environmental pollution and high water consumption [7,8]. Under current climatic conditions, soybeans already pose an environmental sustainability challenge; projections for 2050 suggest that climate-driven production variability will be further amplified by global market dynamics, leading to heightened price volatility [9].

Within this global context, the European Union faces major challenges in maintaining protein self-sufficiency. Approximately 24% of the 71 million tons of crude protein required annually by the EU livestock sector is imported. This dependence rises to 66% for high-protein feed ingredients and reaches 96% for soybean meal [10]. As a result, the European livestock sector is structurally exposed to global market volatility and sustainability concerns associated with soybean production.

This structural dependence is particularly critical for the pig sector, which relies heavily on soybean meal as its primary protein source. In Belgium, soybean meal remains the dominant protein ingredient in pig feed formulations [11], making the sector highly vulnerable to fluctuations in global soybean markets and increasingly exposed to environmental and regulatory constraints linked to deforestation and carbon footprints.

At the regional level, this vulnerability appears in Wallonia. Modeling exercises suggested that full food self-sufficiency is not achievable under current dietary and farming practices unless food waste will be significantly reduced and diets will shift toward more local based options [12].This emphasizes the urgent need for innovative strategies to improve the resilience of regional food systems, such as valorizing agro-industrial by-products and developing alternative protein sources.

Among emerging alternative protein sources to soybean meal, the black soldier fly (BSF), *Hermetia illucens* (L. 1758), has gained increasing attention [13]. This insect, native to tropical regions of South America, is characterized by incredibly voracious, polyphagous larvae, offering the potential to valorize a wide range of unsold products and agro-industrial by-products into high-quality protein [14,15]. BSF larvae are considered a high-quality protein source, as their crude protein content on a dry matter basis ranges around 40%, depending on the rearing substrate, and their amino acid profile is characterized by a relatively favourable proportion of essential amino acids [16,17]. BSF proteins have already demonstrated their potential in pig production by partially replacing conventional protein sources in pig diets [18].

In pigs, the post-weaning period is a critical and delicate stage, requiring a high-quality diet that meets the animal’s physiological needs while limiting the impact of stressors and the occurrence of post-weaning disorders [19]. Studies evaluating the inclusion of BSF proteins in piglet diets, therefore, provide a relevant framework for assessing the nutritional quality of this novel protein source [20,21,22]. However, these trials were not conducted under organic farming conditions. They relied either on synthetic amino acid supplementation or on the inclusion of animal-derived proteins such as whey or fishmeal (FM), raising concerns about feed–food competition and pressure on marine resources. For this reason, an organic pig production system that uses neither traditional animal-derived proteins nor synthetic amino acids provides a particularly relevant model for evaluating the nutritional value of BSF proteins.

The aim of this study was to evaluate whether locally available unsold organic products and agro-industrial by-products in Wallonia can be valorized by BSF larvae and used to replace part of the protein core in organic post-weaning piglet diets with defatted BSF meal. It was hypothesized that moderate inclusion levels of defatted BSF meal would sustain growth performance and feed efficiently similarly to those achieved with an organic soybean-based protein core.

## 2. Materials and Methods

Registrations for insect producers other than bees and bumblebees, as well as for the production of co-products for animal feed, were submitted to the Federal Agency for the Safety of the Food Chain (AFSCA). In addition, the Walloon Agricultural Research Centre (CRA-W; Gembloux, Belgium), which provided the animals, held the required authorizations for pig farming, animal transport (short journeys), and the manufacture of compound feeds, including feeds containing processed animal proteins derived from insects, in accordance with Belgian and European regulations. Approval as a processor of type 3 animal by-products was granted by the Walloon Region. The Ethics Committee of the University of Liège approved the experimental protocol for agricultural experimentation (no. 24-2762).

### 2.1. Larval Production

Unsold organic food residues and brewers’ grain were collected once or twice weekly over a 9-month period from a local organic food store and a brewery. In total, 4730 kg of fresh substrate was collected, consisting of fruits and vegetables (2729 kg), brewers’ grain (1678 kg), and bread (323 kg).

After collection, batches of 89.24 ± 10.76 kg fresh substrate (minimum: 59 kg; maximum: 105 kg) were processed using a digester (Biowaste Upcycling, Waterloo, Belgium). The substrate was ground and heated at 50 °C. Although the standard treatment duration is 24 h, processing was extended to 72 h to obtain a more stable and storable flour-like product [23].

Larvae were reared in plastic tanks (65 × 45 × 15 cm) dedicated to insect production. The initial biomass of 12,000 five-day-old larvae per tank was estimated from the average weight of a subsample of 150 larvae. A total of 414 tanks were used during the production period. Larvae were fed with a reconstituted substrate prepared from 2.4 kg of processed substrate flour mixed with water to obtain a final quantity of 6.860 kg of feeding substrate.

Larvae were maintained in a controlled rearing room at 25.3 ± 0.5 °C and 57.5 ± 14.7% relative humidity at the Functional and Evolutionary Entomology Laboratory of Gembloux Agro-Bio Tech (University of Liège). Larvae were harvested after 11 days of development, before the onset of blackening. Harvested larvae were frozen at −20 °C, mechanically crushed (Steel Fruit Crusher 1100 W, WilTec, Eschweiler, Germany), and dried at 70 °C for 8 h. This process yielded 555 kg of fresh larvae biomass, corresponding to an approximate fresh biomass conversion rate of 11.7% relative to the collected fresh substrate.

### 2.2. Supercritical CO_2_ Extraction Procedure

Defatting of BSF larval meal was performed using a 400 L supercritical CO_2_ extractor (Extratex S.F.I., Pont-Saint-Vincent, France). A total of 147.5 kg of dry meal was processed under constant operating conditions of 60 °C and 600 bar. The CO_2_ mass flow rate was maintained at 16.67 kg min^−1^ (≈1000 kg h^−1^), resulting in a total CO_2_ consumption of 5000 kg over the 6 h extraction period. This corresponded to a solvent-to-feed ratio (S/F) of 34 kg CO_2_ per kg dry matter. The extractor was operated in fixed-bed mode. Lipids were recovered in two cyclonic separators in series, operated at 55 °C and 55 bar (first separator) and 20 °C and 55 bar (second separator). The process yielded in a lipid extraction rate of 28.8%. After extraction, the defatted insect powder was collected, sealed in airtight containers, and stored at −20 °C until further analysis (Table 1).

### 2.3. Sanitary Treatment

The defatted insect protein powder was subsequently subjected to a heat treatment at 80 °C for 120 min, followed by 100 °C for 60 min, in accordance with processing method No. 5 for processed animal proteins as defined by Regulation (EC) No. 1069/2009 and its implementing Regulation (EU) No. 142/2011 [24,25]. The insect meal (Table 1) was then finely ground with a hammer mill (SM 100, Retsch GmbH, Germany) equipped with a 3 mm sieve.

**Table 1:** Composition of insect meal (*Hermetia illucens*) obtained after being defatted with supercritical CO_2_ and composition of the other ingredients comprising the protein core of the feed for post-weaning piglets. Sugar + starch = sum of starch and soluble sugars; NEv’97 = net energy INRA version 1997; Lys = Lysine; Met = Methionine; Thr = Threonine; Trp = Tryptophane; Trp cor = value corrected based on [26]; SID = standard ileal digestibility.

Ingredient	Insect Meal	Organic Soybean Meal	Organic Peas Meal	Potato Protein Meal	Soybean Oil
Crude protein (%)	51.20	47.00	21.20	75.00	0.00
NEv97 (kcal)	2797.00	2368.00	2274.00	2094.00	8124.00
Crude lipids	5.56	7.50	1.20	0.00	99.50
Cellulose (%)	9.22	7.20	5.50	0.00	0.00
Sugars + starch (%)	0.00	13.80	47.10	3.30	0.00
Calcium (%)	4.73	0.27	0.07	0.30	0.00
Phosphorus (%)	0.03	0.61	0.40	0.22	0.00
Lys (%)	3.18	2.91	1.51	5.85	0.00
Met (%)	0.75	0.66	0.21	1.73	0.00
Thr (%)	2.02	1.83	0.78	4.28	0.00
Trp (%)	0.17	0.61	0.19	1.05	0.00
Trp cor (%)	0.47	0.61	0.19	1.05	0.00
SID Lys (%)	2.75	2.55	1.19	5.21	0.00
SID Met (%)	0.69	0.58	0.15	1.55	0.00
SID Thr (%)	1.65	1.51	0.54	3.63	0.00
SID Trp (%)	0.14	0.52	0.12	0.83	0.00
SID Trp cor (%)	0.41	0.52	0.12	0.83	0.00

### 2.4. Chemical Analysis of BSF Larva Meals and Experimental Diets

Before the feeding trial, nitrogen and amino acid composition were determined on the insect meal in order to characterize the ingredient used for diet formulation and to support the estimation of standardized ileal digestible amino acid values.

Nitrogen content was determined using the Kjeldahl method [27] on 0.4 g of sample, using the same analytical equipment and reagents as previously described [23]. Crude protein content was calculated using a nitrogen-to-protein conversion factor of 6.25. Although lower conversion factors have been proposed for insect-derived ingredients [28], the conventional factor of 6.25 was retained in the present study to ensure methodological consistency across all dietary ingredients and experimental diets. As diet formulation was based on standardized ileal digestible (SID) amino acids rather than crude protein values, crude protein data are provided for compositional comparison only.

Amino acid profiling was performed using three parallel hydrolyses: acid hydrolysis for total amino acids, performic acid oxidation followed by hydrolysis for sulfur-containing amino acids, and alkaline hydrolysis for tryptophan. To hydrolyze the samples and release protein-bound amino acids, 6 N HCl containing 0.1% phenol was added to each sample (5 g of insect flour), followed by acid hydrolysis at 110 °C for 24 h under nitrogen to prevent oxidation. After hydrolysis, the samples were adjusted to pH 2.2 before injection into the Biochrom 30 HPLC system (Cambridge, UK). Amino acid separation was performed using a cation-exchange resin column (High performance sodium oxidized column, U-P Resin n°: 09238). Elution was achieved by gradually increasing the pH and ionic strength of the elution buffers and raising the column temperature. Post-column derivatization with ninhydrin was used for detection, and absorbance was measured at 570 nm for general amino acids and at 440 nm for proline [29].

To quantify sulfur-containing amino acids (methionine and cysteine), performic acid oxidation was performed before acid hydrolysis. The cooled samples were treated with an ice-cold performic acid mixture (formic acid, phenol, and hydrogen peroxide) and allowed to react for 16 h at 4 °C. Excess reagent was neutralized with sodium metabisulfite, and the samples were then subjected to standard acid hydrolysis as described above. After derivatization, cysteine and methionine were determined as cysteic acid and methionine sulfone, respectively [30].

Tryptophan, which degrades during acid hydrolysis, was quantified separately following alkaline hydrolysis. Samples were treated with 4 M barium hydroxide and hydrolyzed for 20 h at 110 °C under nitrogen. The hydrolysates were neutralized with sodium acetate buffer (pH 4.25), spiked with α-methyltryptophan (2.5 µM/mL) as an internal standard, filtered, and analyzed by HPLC-UV using a Waters Xterra RP18 column (150 × 4.6 mm, 3.5 µm). Elution was performed with a sodium acetate–methanol–acetonitrile gradient, and detection was at 280 nm [31]. All amino acid concentrations were expressed as grams per 100 g of fresh matter.

After the feeding trial, total amino acid composition was analyzed on the experimental diets using acid hydrolysis followed by HPLC analysis according to the laboratory protocol. As described in the analytical procedure, feed samples were hydrolyzed with 6 N HCl containing 0.1% phenol at 110 °C for 24 h, then diluted, filtered (0.22 µm), derivatized using the AccQ-Tag reagent system (Waters Corporation, Milford, MA, USA), and analyzed by HPLC. The protocol also included external standard preparation (Amino Acid Standard H, Thermo Scientific, Waltham, MA, USA) a defined elution gradient, and concentration calculations based on the overall dilution factor of the sample preparation procedure [32].

Ash content was determined by incinerating 0.8 g of the sample, increasing the temperature by 50 °C every 30 min until reaching 550 °C, then holding it at 550 °C for 4 h [33]. Mineral profiling (phosphate, calcium, potassium, magnesium, and sodium) was performed by mineralizing 0.5 g of the sample in 5 mL of aqua regia [1:3 *v*/*v* (nitric acid: hydrochloric acid)] for 2 h under reflux. The concentrations of these elements were measured using an atomic absorption spectrometer (AAS) (PerkinElmer AAnalyst 200^®^, Waltham, MA, USA) [34].

Lipid content was determined using a Soxhlet apparatus according to the AOAC method 945.1 [35].

Experimental diets were formulated for organically reared post-weaning piglets over 5 weeks, based on the PRODABIO (Awans, Belgium) starter piglet energy and protein core. Four isoenergetic and isonitrogenous diets were designed (Table 2), consisting of a fixed energy core representing 81.2% of the diet and a variable protein core (18.8%). The energy core was consistent across all treatments, whereas the protein core composition differed by partially replacing conventional protein sources with insect meal (*H. illucens* defatted using supercritical CO_2_ extraction).

The control diet contained no insect meal and included only the commercial protein base. The three experimental diets contained 15%, 25%, and 35% insect meal, respectively, in their protein core. The basal protein core consisted of organic soybean meal 46, organic pea flour, and potato protein, supplemented with organic soybean oil.

All diets were formulated to be isoenergetic (net energy according to INRA, 1997) and isonitrogenous by adjusting the protein core composition to maintain constant standardized ileal digestibility levels for lysine, methionine, threonine, and tryptophan (Table 3). Insect meal digestibility coefficients used for formulation were obtained from the literature [26].

### 2.5. Animal Care and Management Procedures

After birth at CRA-W the piglets received standard care in accordance with organic farming regulations. Sanitel ear tags were applied on the left ear, teeth clipping and tail docking were performed, and iron supplementation (Uniferon) was administered. No anticoccidial treatment (Dozuril) was provided. Piglets were vaccinated with Cyrcomax Myco (Zoetis, Louvain-la-Neuve, Belgium) at 4 weeks of age against *Mycoplasma hyopneumoniae* and porcine circovirus. Peat was supplied as bedding material, and a standard control creep feed was offered from the second week of age. Some sows that experienced difficulties during farrowing had to receive antibiotics.

Seventy-two female crossbred piglets [Landrace × Piétrain] were weaned at 34 days old and transported to the Animal Production Centre of Gembloux Agro-Bio Tech (CEPA-University of Liège, Gembloux, Belgium) for 5 weeks of post-weaning rearing. The seventy-two weaned piglets were assigned to four treatments. Each treatment was distributed across six pens (1.5 m^2^, with polymer grating, a heat lamp, and enrichment with steel chains), each containing three piglets.

During the trial, after one week, one piglet from group 4 was temporarily removed from its pen because of a minor leg injury and housed individually for two weeks under veterinary supervision. After recovery, the piglet was returned to its original pen and completed the experiment. Because the experimental unit was the pen, and group composition was restored thereafter, this event was not considered to affect the integrity of the dataset, and all pens were retained in the analyzes.

### 2.6. Statistical Analysis

All analyses were performed at the pen level (n = 18; 3 pigs per pen). Body weight was recorded on days 0, 7, 14, 21, 28, and 35. Weekly feed intake was measured per pen and matched with the corresponding weekly interval; feed intake at week 0 was not applicable and was recorded as missing.

Two longitudinal mixed-effects models were fitted with restricted maximum likelihood (REML) using the lme function in the nlme package (v. 3.1.168) [36]. Time was modelled as a continuous variable (“Week”). Pen was the random grouping factor. In all models, we considered a first-order autoregressive AR(1) within-pen correlation structure across weeks and allowed for heteroscedastic residual variance by week using varIdent. Competing error structures (independence, compound symmetry, AR(1); with/without heteroscedasticity) were compared by Akaike’s Information Criterion (AIC), and the structure with the lowest AIC was retained.

Weekly gain (kg/pen/week) was defined as the week-to-week difference in pen body weight; analyses were restricted to weeks ≥ 1. Fixed effects were Diet (4 levels; 1 = control), Week, Lateral position of the pen in the room (Left/Right), Longitudinal position (Entrance/Middle/Back), and Weekly feed consumption (kg/pen/week), as covariates. The pen random effect was a random intercept. The retained error structure was AR(1) with week-specific residual variances.

Pen body weight (kg) across weeks 0–5 was modelled using fixed effects for Diet, Week, their interaction (Diet × Week), and the two spatial factors (Lateral and Longitudinal positions). The pen random effect was a random intercept. As above, an AR(1) correlation and week-specific residual variances were used. (For numerical stability, Week was mean-centred in this model; results are reported at the original week values via marginal means.)

Model assumptions were checked graphically (residuals vs. fitted values and normal Q–Q plots). Missing values were handled using listwise deletion during model fitting (na.action = na.omit). Estimated marginal means (EMMs) and pairwise contrasts by diet at each week were obtained with emmeans (emmeans package, v. 1.11.2.8), with Tukey multiplicity adjustment [37]. For the gain model, the EMMs and contrasts were computed at the mean weekly consumption and modal levels of the spatial factors. Effects are presented as estimates with 95% confidence intervals; two-sided α = 0.05 was used for significance.

### 2.7. Cost Model and Break-Even Price

To translate performance into economic terms, adjusted feed conversion ratios (FCR) per diet were calculated at a representative weekly gain, and the break-even price of insect protein that yields the same feed cost per kg of liveweight gain as the control was derived.

Adjusted FCRs. From the fitted “feed vs. gain” mixed model (see previous subsection), we obtained, for each diet, the predicted weekly feed intake at a representative weekly gain g_”ref” (the sample median), with covariates fixed to typical values (modal Lateral/Longitudinal positions, mean initial pen weight, representative week). The adjusted mean FCR was then

$${FCR}_{r}^{\mathrm{mean},\mathrm{adj}}\;=\;\frac{{\hat{Feed}}_{r}\left(g_{\mathrm{ref}}\right)}{g_{\mathrm{ref}}}\left(\mathrm{kg}\;\mathrm{feed}\;\mathrm{per}\;\mathrm{kg}\;\mathrm{gain}\right),$$

where r indexes diet. Estimated marginal means were obtained with the emmeans package.

Diets consisted of an energy core (price (P_e = €529)·t^−1^, excluding value-added tax (VAT); share (f_e = 0.812)) and a protein core (control price (P_0 = €1039)·t^−1^, excluding VAT; share (f_p = 0.188)). These prices corresponded to the actual feed prices charged by suppliers during the experimental period. The control feed cost per ton was

$$C_{0}\;=\;f_{e}P_{e}+f_{p}P_{0}\;\approx \frac{\;€625}{t}.$$

In insect diets, a fraction of the protein core was replaced with insect protein at a price (€/t). The energy core was unchanged. The insect protein break-even price X_”break” equates feed cost per kg gain between the insect diet and control:

$$X_{\mathrm{break}}\;=\;P_{0}\;+\;\frac{\left(\frac{{FCR}_{0}^{\mathrm{mean},\mathrm{adj}}}{{FCR}_{r}^{\mathrm{mean},\mathrm{adj}}}-1\right)\;C_{0}}{f_{p}\;r},$$

where ${FCR}_{0}^{\mathrm{mean},\mathrm{adj}}$ and ${FCR}_{r}^{\mathrm{mean},\mathrm{adj}}$ are the adjusted FCRs for the control and diet r, respectively. If the market price $X\leq X_{\mathrm{break}}$, incorporating insect protein at a rate *r*, does not increase the feed cost per kg gain. As a robustness check, we computed approximate 95% CIs $X_{\mathrm{break}}$ via a delta method propagating the uncertainty in the adjusted FCRs.

## 3. Results

### 3.1. Diet Analyses

The chemical analyses of the experimental diets confirmed that they were comparable across treatments in terms of dry matter, organic matter, nitrogen, crude protein, neutral detergent fiber, ether extract, and amino acid profiles (Table 4).

### 3.2. Weekly Gain

**Figure 1:**
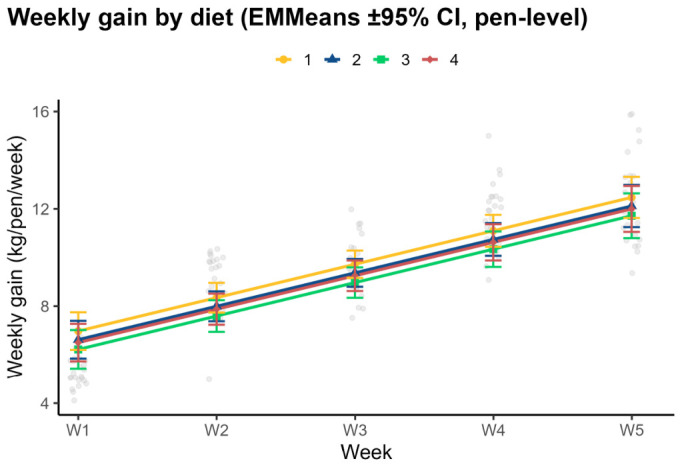
Model-adjusted weekly body-weight gain (kg per pen per week) by diet in post-weaning piglets. Lines and error bars represent estimated marginal means (EMMeans ± 95% confidence intervals) obtained from a linear mixed-effects model with pen as a random intercept, AR(1) temporal correlation, week-specific residual variances, and dry matter intake included as a covariate. Points show observed pen-level weekly gains. Model predictions are displayed for weeks 1 to 5 at the mean dry matter intake and modal spatial pen positions.

Weekly gain (kg/pen/week) was analyzed using a linear mixed-effects model with pen as a random intercept, an AR(1) within-pen correlation structure, and week-specific residual variances (varIdent). Allowing heterogeneous residual variances improved model fit compared with a homoscedastic AR(1) model (AIC = 226.8 vs. 229.2) and the estimated AR(1) parameter indicated a moderate positive within-pen correlation (ϕ ≈ 0.30). After adjustment for covariates, the weekly gain did not vary linearly across weeks, and the week effect was retained for temporal structure only. Compared with the control diet (R1), weekly gain was lower for R3 (−0.75 kg/pen/week, SE ≈ 0.32, *p* = 0.048), whereas no significant difference was detected between R1 and either R2 or R4 (*p* > 0.10). Dry matter (DM) intake was strongly associated with weekly gain, with an estimated increase of approximately +0.16 kg gain per additional kg DM intake per pen per week (*p* < 0.001). No significant effect of lateral or longitudinal pen position was detected (*p* > 0.05). Because no interaction between diet and week was included by design; diet effects represent average differences across weeks. Estimated marginal means of weekly gain, computed at mean DM intake and modal spatial positions, increased over time as expected (Figure 1) and. Tukey-adjusted pairwise contrasts between diets were not significant at any individual week (*p* > 0.05). Although weekly gain tended to be lower for R3 on average across the study period, week-specific contrasts were not significant after Tukey adjustment. This likely reflects the relatively high week-to-week variability in gain measurements and the fact that the weekly gain model estimated average diet effects across weeks without including Diet × Week interactions. In contrast, the body-weight trajectory model evaluated cumulative growth over time and allowed diet-specific slopes, such that relatively small but repeated differences in weekly gain progressively accumulated and became detectable at later weeks.

### 3.3. Body Weight Trajectories

Pen body weight (kg) across weeks 0 to 5 was analyzed using a linear mixed-effects model with a pen-level random intercept, an AR(1) correlation structure, and week-specific residual variances. This model provided a better fit than the corresponding generalized least squares model without a random effect (AIC LME = 548.9 vs. GLS = 561.3). The AR(1) parameter was high (ϕ = 0.989), indicating strong temporal dependence. Residual variance increased with week (relative residual standard deviations: 1.00, 0.55, 1.37, 2.34, 3.92, and 5.69) and a strong main effect of week (centred) was observed (+6.96 ± 0.16 kg/pen/week, *p* < 0.001).

At the reference week, the main effects of diet relative to R1 were −1.73 ± 0.75 kg for R3 (*p* = 0.033), −0.36 ± 0.75 kg for R2 (*p* > 0.05), and −1.27 ± 0.75 kg for R4 (*p* > 0.05). Significant Diet × Week interactions were detected for R3 (−0.60 ± 0.22 kg/week, *p* = 0.007) and R2 (−0.47 ± 0.22 kg/week, *p* = 0.034), whereas the interaction for R4 was not significant (*p* > 0.05). A negative trend was observed for lateral pen position (−0.92 kg, *p* = 0.056).

**Figure 2:**
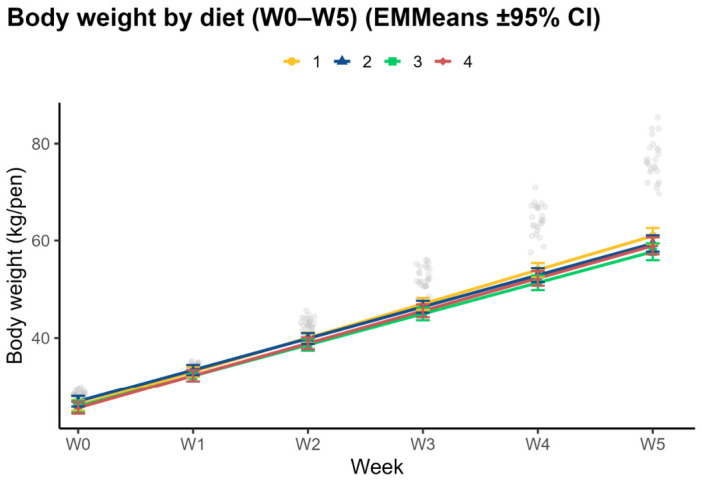
Model-adjusted body weight trajectories (kg per pen) throughout the experimental period (weeks 0–5) by diet. Lines and error bars represent estimated marginal means (EMMeans ± 95% confidence intervals) derived from a linear mixed-effects model including pen as a random intercept, an AR(1) correlation structure, and week-specific residual variances, with spatial pen positions included as fixed effects. Points represent observed pen-level body weights. Model predictions illustrate diet-specific growth trajectories over time.

Because body weight represents the cumulative result of successive weekly gains, relatively small differences in gain efficiency between diets became progressively amplified over time, leading to divergence in predicted body weight trajectories. Estimated marginal means (averaged across spatial positions) indicated divergence among diets over time. At week 5, predicted pen body weights were 60.6 kg for R1, 59.1 kg for R2, 57.4 kg for R3, and 58.6 kg for R4 (Figure 2). The difference between R1 and R3 at week 5 was significant (3.23 kg; Tukey-adjusted *p* = 0.042), whereas other between-diet contrasts at individual weeks were not significant (*p* > 0.05).

### 3.4. Weekly Feed Conversion Ratio (FCR)

**Figure 3:**
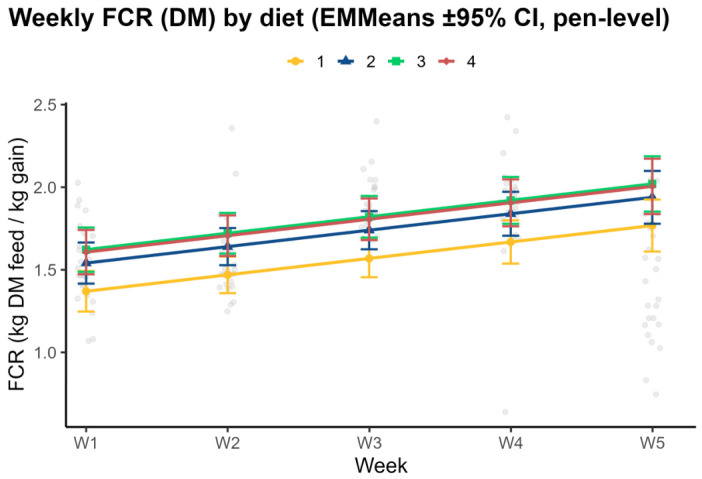
Model-adjusted weekly feed conversion ratio (FCR; kg DM feed per kg body weight gain) by diet in post-weaning piglets. Lines and error bars represent estimated marginal means (EMMeans ± 95% confidence intervals) derived from a linear mixed-effects model including pen as a random intercept, an AR(1) within-pen correlation structure, and week-specific residual variances, with spatial pen positions included as fixed effects. Points represent observed pen-level weekly FCR values. Estimates are shown for weeks 1 to 5.

Weekly feed conversion ratio (FCR), calculated as DM intake divided by weekly gain at the pen level, was analyzed using a mixed-effects model with a random intercept per pen, AR(1) correlation, and week-specific residual variances. Diet had a significant overall effect on weekly FCR (*p* ≈ <0.013). Compared with the control diet (R1), weekly FCR was higher for R3 (+0.23 ± 0.08, *p* ≈ <0.012) and R4 (+0.21 ± 0.08, *p* ≈ <0.018), whereas the difference between R2 and R1 was not statistically significant (*p* > 0.05). These results indicate that higher inclusion levels of insect meal (R3 and R4) slightly reduced weekly feed efficiency, whereas a 15% replacement (R2) maintained FCR comparable to that of the control diet. No significant effects of spatial position were detected. Model-adjusted estimated marginal means of weekly FCR across weeks are shown in Figure 3.

### 3.5. Cumulative Feed Conversion Ratio

Cumulative feed conversion ratio (FCR_total_DM), defined as total DM intake divided by total body weight gain from week 1 to week 5 at the pen level, did not differ significantly among diets (ANOVA Diet effect: *p* = 0.101). Mean cumulative FCR values (±SEM) were 1.57 ± 0.07 for R1, 1.66 ± 0.05 for R2, 1.68 ± 0.06 for R3, and 1.72 ± 0.06 for R4. Tukey-adjusted pairwise comparisons were not significant (*p* > 0.05). Despite the significant diet effect on weekly FCR, these differences did not translate into statistically significant changes in cumulative FCR over the 5-week period.

### 3.6. Economic Break-Even Analysis (DM Basis)

Using cumulative feed conversion ratio calculated on a dry matter basis over weeks 1–5 and a protein nucleus fraction of f_p_ = 0.188, break-even prices for the insect-derived ingredient were calculated relative to the control protein nucleus price (P_0_ = €1039·t^−1^). Based on the observed cumulative DM-based FCR, the break-even price was negative for the 15% inclusion level (R2: −€133·t^−1^) and remained well below the control protein nucleus price at higher inclusion levels (R3: €131·t^−1^; R4: €214·t^−1^). These results indicate that, under the observed feed efficiency, inclusion of defatted BSF meal could not achieve economic parity with the control diet at any realistic market price.

## 4. Discussion

European pig production faces a structural deficit in locally available protein-rich feedstuffs, which increases dependence on imported soybean meal, and exposure to international price volatility and sustainability concerns [9]. In contrast to alternative protein crops, BSF can convert unsold and agro-industrial co-products into high-quality protein, thereby coupling feed production with waste reduction and circularity [13]. This experiment demonstrated the feasibility of processing several tons of unsold local organic products and by-products into BSF protein and conducting a five-week organic feeding trial on post-weaned piglets by partially replacing the protein core of the diet with defatted BSF meal.

Our findings showed that a 15% replacement of the protein core with defatted BSF meal (diet 2) resulted in growth performance (weekly gain, total gain, and feed conversion ratio) comparable to that of the control diet, whereas higher inclusion rates (25% and 35%) led to slightly but significantly reduced performances. Although the 25% inclusion rate numerically showed lower values than the 35% inclusion rate for some parameters, these differences were not statistically significant and therefore cannot be interpreted as evidence of a specific underperformance at the intermediate inclusion level. These results are consistent with the literature, which reports that low inclusion levels of BSF generally maintain performance similar to that of control diets, whereas higher inclusion levels tend to compromise growth efficiency, especially in the absence of amino acid supplementation or animal-derived proteins, as required by biological agriculture. Moreover, the diets used in this trial did not contain animal-derived proteins such as whey or fishmeal, which are known to improve amino acid balance and palatability.

Nevertheless, our findings are in partial agreement with those of [38], who reported that including 12.5% of defatted BSF meal in the total diet maintained growth performance comparable to that of a control diet containing fishmeal, whey, and spray-dried porcine plasma. Although the phase during which piglets were fed exclusively on solid feed began one week earlier, feed conversion ratio (FCR) values ranged between 1.67 and 2.16, which are comparable to those obtained with diets containing 21.01% and 20.65% crude protein and metabolizable energy levels of 4100 and 4070 kcal/kg, respectively. However, they reported reduced performance at a 25% inclusion rate, which may be attributed to the decline in animal-derived protein sources that provide a more optimal amino acid profile for piglets. Similarly, good performance was reported [39] with a 12.0% inclusion of full-fatted BSF meal in a diet providing 3265 kcal/kg and 21.5% crude protein, containing 6.0% whey powder and supplemented with lysine. The inclusion of up to 19.0% BSF meal resulted in only minor effects on performance when replacing fishmeal and part of the soybean meal in a diet providing 2502 kcal/kg net energy and 20.7% crude protein, supplemented with lysine, methionine, and threonine [40]. Several insect species have been investigated, and inclusion levels below 10% of BSF, *Tenebrio molitor* L. 1758, or *Alphitobius diaperinus* Panzer, 1797 in post-weaning diets supplemented with essential amino acids produced performance comparable to that obtained with enzyme-treated soybean meal during the first two weeks after weaning [41]. These results indicate that higher inclusion rates are easier to achieve in conventional systems, where amino acid deficiencies can be corrected through targeted supplementation.

In this experiment, diets were formulated to be isoenergetic and isonitrogenous, with constant SID values for key amino acids. Chemical analyses further confirmed that the diets were comparable across treatments. However, these SID coefficients were theoretical and not directly measured, and they do not account for the full spectrum of indispensable and dispensable amino acids nor their potential interactions. Differences in SID between amino acids from defatted BSF meal and those from the protein core of the diets could therefore explain the performance differences observed in the insect-based diets. If a single amino acid becomes limiting for protein synthesis, it may reduce growth performance in piglets fed diets with high levels of insect meal [42]. In post-weaning piglets, many amino acid imbalances can affect feed intake, weight gain, and feed conversion ratio. Branched-chain amino acids (BCAAs) may reduce feed intake and efficiency when valine is deficient relative to leucine [43]. A low valine-to-lysine ratio can also lead to decreased feed intake and reduced growth performance [44]. Finally, the total amount of digestible indispensable amino acids and their ratio to digestible indispensable amino acids are factors that are rarely considered yet appear to play an important role in diet formulation in general [45]. One possible improvement of the present experiment would be to refine our understanding of the ideal protein requirements of piglets and to formulate diets based on the SID of all amino acids. Such an approach may require modifying the protein core to better complement the amino acid profile of insect meal.

The results of this experiment are consistent with those reported in the literature and show that BSF proteins can replace part of the traditional protein sources in piglet diets and, more broadly, in pig production [21,38,39,40,46,47]. Although BSF proteins cannot fully replace the entire protein core or soybean meal, their inclusion can partially reduce dependence on imported protein sources and limit feed–food competition or competition for arable land, while maintaining adequate feed performance.

Organic farming still accounts for only a minor share of total agricultural production in Belgium and across the European Union [48]. However, based on our results and the available literature, it can be assumed that in conventional production systems, higher inclusion levels of defatted BSF protein than 15% of the protein core could be achieved through appropriate supplementation with synthetic amino acids [49,50]. These findings are particularly encouraging, as the use of BSF proteins as a replacement for traditional protein sources is not limited to pig production and has already shown promising results in poultry and aquaculture [51,52]. Nevertheless, it is important to keep in mind that, regardless of the animal production system, a complete replacement of the protein core at the same cost and performance is not currently achievable.

While these results highlight the biological feasibility of partial replacement of conventional protein sources by BSF meal, they also emphasize the need to consider economic constraints. In the present study, break-even analysis based on cumulative dry matter–based feed conversion ratio showed that even modest changes in feed efficiency strongly influenced economic outcomes, particularly at low substitution rates. This underlines that the economic relevance of BSF proteins cannot be assessed independently of feed efficiency and inclusion level, thereby justifying a broader discussion of cost drivers and value creation beyond feed formulation alone. Several options can be considered to improve the economic competitiveness of insect proteins, particularly BSF proteins. First, it is essential to consider the full range of services BSF farming provides. This experiment and numerous studies demonstrate that BSF larvae can valorize a wide range of unsold products, agro-industrial by-products, and even biowaste [23,53,54]. By recycling organic matter, BSF larvae produce not only large quantities of protein but also lipids and chitin [55]. The high proportion of saturated fatty acids in BSF lipids can make diet formulation challenging; however, insects can be efficiently defatted [56]. Although large-scale defatting methods, such as supercritical CO_2_ extraction used in the present study, are costly, valorizing BSF lipids may help offset these costs. BSF lipids are already being investigated for applications in ruminant nutrition [57,58] and for industrial uses, such as lubricant additives [59] and biofuels [60,61].

Finally, frass, a mixture of predigested feed residues and insect excreta, can be used as a pre-compost or fertilizer [62]. Beyond valorizing all molecules and co-products generated by BSF production, a second approach to reducing costs would be to develop dedicated insect production chains or integrate BSF farming into existing agricultural value chains. In Belgium and at the European Union scale, BSF farming remains costly, mainly due to energy requirements and regulatory constraints, including those related to insect defatting [63,64,65,66]. Designing regional or large-scale insect production systems with dedicated infrastructure could enable economies of scale and optimize logistics for production and valorization. Finally, simplifying and clarifying European regulations would facilitate the development of insect farming for both organic waste recycling and food and feed production.

## 5. Conclusions

This study demonstrates the feasibility of using black soldier fly larvae to valorize unsold organic products and agro-industrial by-products by replacing 15% of the protein core in organic post-weaning piglet diets over a five-week period in Belgium. This inclusion level represents a realistic and biologically relevant threshold under organic farming conditions, where the use of synthetic amino acids and animal-derived proteins is restricted, while maintaining growth performance and feed efficiency comparable to those of a conventional organic protein core.

Beyond its nutritional value, this approach contributes to the development of circular feed systems by converting locally available organic residues into high-value animal protein, thereby reducing food waste and partially decreasing dependence on imported soybean meal. From an economic perspective, break-even analysis based on cumulative dry matter–based feed conversion ratio indicated that, under the observed feed efficiency, organic defatted BSF meal could not achieve economic parity with conventional organic protein sources at typical market prices when feed cost per kilogram of gain was considered alone.

However, the rationale for integrating BSF into pig production systems extends beyond immediate feed cost reduction and lies primarily in its contribution to circularity. BSF larvae enable biowaste management and the valorization of local organic residues, generate co-products such as lipids and frass with additional market value, and may contribute to improving regional protein self-sufficiency. Consequently, the economic relevance of BSF-derived proteins will depend on substantial reductions in ingredient cost, improvements in feed efficiency, and the structuring of efficient insect production chains, including optimized processing technologies and the valorization of co-products.

## Figures and Tables

**Table 2:** Composition (in%) of protein and energy cores in different diets (1 = 0%; 2 = 15%; 3 = 25%; 4 = 35% replacement of protein core with insect meal) used to feed post-weaning piglets. As well as the measured nutritional value of each diet. Crude protein = nitrogen × 6.25.

Diet Name	1	2	3	4
**Protein core (%)**	**18.80**	**18.80**	**18.80**	**18.80**
Organic soybean meal (%)	63.42	53.91	47.57	30.92
Potato protein (%)	24.80	21.08	18.60	12.09
Organic peas	11.00	9.35	8.25	5.36
Organic soybean oil (%)	0.77	0.65	0.58	0.38
Defatted black soldier fly (%)	0.00	15.00	25.00	35.00
**Energy core (%)**	**81.20**	**81.20**	**81.20**	**81.20**
Organic barley (%)	30.60	30.60	30.60	30.60
Organic wheat (%)	24.50	24.50	24.50	24.50
Organic corn (%)	22.25	22.25	22.25	22.25
Organic fine bran (%)	9.25	9.25	9.25	9.25
Organic triticale (%)	6.25	6.25	6.25	6.25
Organic premix pork–poultry 1.25 (%)	1.54	1.54	1.54	1.54
Chalk (%)	1.43	1.43	1.43	1.43
Calcium monophosphate (%)	1.19	1.19	1.19	1.19
Organic beet pulp (%)	1.00	1.00	1.00	1.00
Organic soybean oil (%)	0.92	0.92	0.92	0.92
Salt (%)	0.69	0.69	0.69	0.69
Organic Elysee (%)	0.24	0.24	0.24	0.24
Levuflor (%)	0.06	0.06	0.06	0.06
Betaine (%)	0.03	0.03	0.03	0.03
Cibus nat arome (%)	0.02	0.02	0.02	0.02
Digestarom pro (%)	0.02	0.02	0.02	0.02

**Table 3:** Nutritional composition of feed for post-weaning piglets g/kg as feed, including their energy and protein contents. MScor corresponds to the feed based solely on its dry matter content. NEv’97 = net energy INRA version 1997; Lys = Lysine; Met = Methionine; Cys = Cysteine; Thr = Threonine; Tryptophane; cor = corrected; SID = standard ileal digestibility.

Fraction of the Feed	Protein Core as Feed (g/kg)	Energy Core as Feed (g/kg)	Piglet Feed as Feed (g/kg)	Piglet Feed MScor (g/kg)
Dry matter	926.49	875.92	885.43	1000.00
Crude protein	507.43	94.79	172.37	194.67
Ether extract	56.56	33.04	37.46	42.31
Crude cellulose	51.71	33.42	36.86	41.63
Crude ashes	47.77	65.26	61.97	69.99
Sugars + starch	147.51	519.32	449.42	507.57
Sugars	62.25	22.56	30.02	33.91
Amylose	80.38	473.73	399.78	451.51
Nitrogen	81.19	16.93	29.01	32.76
Organic dry matter	923.32	827.96	845.89	955.34
Nev’97	2334.35	2230.07	2249.67	2540.77
Calcium	1.86	10.61	8.97	10.13
Phosphorus	4.85	6.32	6.04	6.82
Phosphate	11.70	14.74	14.16	16.00
Digestible phosphorus	2.12	3.04	2.87	3.24
Magnesium	2.02	1.72	1.78	2.01
Sodium	0.23	4.05	3.33	3.76
Linoleic acid	23.86	15.69	17.22	19.45
Chlorine	0.00	6.55	5.32	6.01
Iron	0.00	92.40	75.03	84.74
Lys	34.65	4.20	9.93	11.21
Met + Cys	16.30	4.06	6.36	7.18
Met	8.69	2.33	3.53	3.98
Thr	23.09	3.41	7.11	8.03
Trp	6.69	1.17	2.20	2.49
SID Lys	30.39	2.83	8.02	9.05
SID Met + Cys	13.58	3.77	5.61	6.34
SID Met	7.70	2.14	3.19	3.60
SID Thr	19.18	1.97	5.21	5.88
SID Trp	5.49	0.76	1.65	1.86
Vitamin A	0.00	12,320.00	10,003.84	11,298.28
Vitamin D3	0.00	2464.00	2000.77	2259.66
Vitamin E	0.00	192.50	156.31	176.54

**Table 4:** Chemical composition (dry matter, organic matter, nitrogen, crude protein, neutral detergent fiber, and ether extract) and amino acid profile of the experimental diets. Analyses were performed on one pooled composite sample per dietary treatment, analyzed in technical duplicate; therefore, no standard deviation is reported.

Diet Names	1	2	3	4
Dry matter %	89.16	89.30	89.76	89.76
Organic matter %	83.34	83.59	83.65	83.67
Nitrogen %	2.79	2.76	2.85	2.91
Crude protein %	17.42	17.25	17.83	18.21
Neutral detergent fiber %	16.32	17.00	16.95	17.85
Ether extract %	3.48	3.31	3.33	3.13
Amino acids g/100 g	16.42	15.90	16.74	15.85
Asp g/100 g	1.46	1.42	1.51	1.41
Ser g/100 g	0.89	0.84	0.86	0.78
Glu g/100 g	2.74	2.78	2.95	2.72
Gly g/100 g	0.85	0.82	0.88	0.84
His g/100 g	0.48	0.47	0.49	0.47
Arg g/100 g	1.14	1.07	1.08	1.04
Thr g/100 g	0.78	0.72	0.74	0.69
Ala g/100 g	0.78	0.81	0.89	0.88
Pro g/100 g	1.16	1.16	1.24	1.18
Cys g/100 g	0.18	0.17	0.15	0.13
Tyr g/100 g	0.54	0.52	0.56	0.55
Val g/100 g	0.96	0.93	0.99	0.97
Met g/100 g	0.15	0.17	0.20	0.16
Lys g/100 g	1.00	0.94	1.00	0.94
Ile g/100 g	0.84	0.79	0.84	0.81
Leu g/100 g	1.50	1.41	1.46	1.39
Phe g/100 g	0.97	0.90	0.92	0.87

## Data Availability

The original contributions presented in this study are included in the article/Supplementary Materials (Data S1). Further inquiries can be directed to the corresponding authors.

## Supplementary Materials

The following supporting information can be downloaded at: https://www.mdpi.com/article/10.3390/insects17060584/s1, Data S1: Raw data from the 5-week post-weaning piglet feeding trial. Regime indicates dietary treatment (1 = control diet, 2 = 15% insect protein replacement in the protein core, 3 = 25% replacement, and 4 = 35% replacement). Weighing day corresponds to weekly body weight measurements. Body weight is expressed in kg. Feed intake represents feed consumption per pen (3 piglets per pen). Lateral position indicates pen location within the room (L = left side, R = right side). Longitudinal position indicates pen location along the room axis (D = near the door).

## Abbreviations

The following abbreviations are used in this manuscript:

BSF	Black Soldier Fly
FM	Fishmeal
AFSCA	Federal Agency for the Safety of the Food Chain
CRA-W	Walloon Agricultural Research Centre
DM	dry matter
Cor	Corrected
SID	standard ileal digestibility
AIC	Akaike’s Information Criterion
REML	restricted maximum likelihood
AR	Autoregressive model
EMMs	Estimated marginal means
FCR	feed conversion ratios
LME	Linear Mixed Effects

## References

[1] Parrini S., Aquilani C., Pugliese C., Bozzi R., Sirtori F., Parrini S., Aquilani C., Pugliese C., Bozzi R., Sirtori F. (2023). Soybean Replacement by Alternative Protein Sources in Pig Nutrition and Its Effect on Meat Quality. Animals.

[2] Sońta M., Rekiel A., Więcek J., Batorska M., Puppel K., Sońta M., Rekiel A., Więcek J., Batorska M., Puppel K. (2021). Alternative Protein Sources vs. GM Soybean Meal as Feedstuff for Pigs—Meat Quality and Health-Promoting Indicators. Animals.

[3] USDA Production—Soybeans 2025. https://www.fas.usda.gov/data/production/commodity/2222000.

[4] Gibbs H.K., Rausch L., Munger J., Schelly I., Morton D.C., Noojipady P., Soares-Filho B., Barreto P., Micol L., Walker N.F. (2015). Brazil’s Soy Moratorium. Science.

[5] Marin F.R., Zanon A.J., Monzon J.P., Andrade J.F., Silva E.H.F.M., Richter G.L., Antolin L.A.S., Ribeiro B.S.M.R., Ribas G.G., Battisti R. (2022). Protecting the Amazon Forest and Reducing Global Warming via Agricultural Intensification. Nat. Sustain..

[6] Peng D., Zhang H., Zhang Y., Yu L., Chen M., Chen J.M., You L., Li P., Liu J., Zhang X. (2026). Global Soybean Trade Dynamics: Drivers, Impacts, and Sustainability. Innovation.

[7] Dreoni I., Matthews Z., Schaafsma M. (2022). The Impacts of Soy Production on Multi-Dimensional Well-Being and Ecosystem Services: A Systematic Review. J. Clean. Prod..

[8] Zhang Q., Hong J., Zhang T., Tian X., Geng Y., Chen W., Zhai Y., Liu W., Shen X., Bai Y. (2023). Environmental Footprints of Soybean Production in China. Environ. Dev. Sustain..

[9] Qiao C., Cheng C., Ali T. (2023). How Climate Change and International Trade Will Shape the Future Global Soybean Security Pattern. J. Clean. Prod..

[10] European Commission (2024). Can the EU Chart a Sustainable Transition to Greater Protein Self-Sufficiency?. https://joint-research-centre.ec.europa.eu/jrc-news-and-updates/can-eu-chart-sustainable-transition-greater-protein-self-sufficiency-2024-10-08_en.

[11] Mierlo K.V., Baert L., Bracquené E., Tavernier J.D., Geeraerd A., Mierlo K.V., Baert L., Bracquené E., Tavernier J.D., Geeraerd A. (2021). The Influence of Farm Characteristics and Feed Compositions on the Environmental Impact of Pig Production in Flanders: Productivity, Energy Use and Protein Choices Are Key. Sustainability.

[12] Desmarez T., Bindelle J., Dumont B. (2025). Towards Sustainable Diets and Farming Systems through Land Use Optimisation. npj Sustain. Agric..

[13] Wang Y.-S., Shelomi M., Wang Y.-S., Shelomi M. (2017). Review of Black Soldier Fly (*Hermetia illucens*) as Animal Feed and Human Food. Foods.

[14] Magee K., Halstead J., Small R., Young I., Magee K., Halstead J., Small R., Young I. (2021). Valorisation of Organic Waste By-Products Using Black Soldier Fly (*Hermetia illucens*) as a Bio-Convertor. Sustainability.

[15] Luttenschlager H., Beckers Y., Francis F., Caparros Megido R. (2026). Meeting Livestock Protein and Amino Acids Needs With Edible Insects: A Critical Review. Sustain. Food Proteins.

[16] Spranghers T., Ottoboni M., Klootwijk C., Ovyn A., Deboosere S., Meulenaer B.D., Michiels J., Eeckhout M., Clercq P.D., Smet S.D. (2016). Nutritional Composition of Black Soldier Fly (*Hermetia illucens*) Prepupae Reared on Different Organic Waste Substrates. J. Sci. Food Agric..

[17] Fuso A., Barbi S., Macavei L.I., Luparelli A.V., Maistrello L., Montorsi M., Sforza S., Caligiani A., Fuso A., Barbi S. (2021). Effect of the Rearing Substrate on Total Protein and Amino Acid Composition in Black Soldier Fly. Foods.

[18] Lu S., Taethaisong N., Meethip W., Surakhunthod J., Sinpru B., Sroichak T., Archa P., Thongpea S., Paengkoum S., Purba R.A.P. (2022). Nutritional Composition of Black Soldier Fly Larvae (*Hermetia illucens* L.) and Its Potential Uses as Alternative Protein Sources in Animal Diets: A Review. Insects.

[19] O’Doherty J., Dowley A., Conway E., Sweeney T., O’Doherty J., Dowley A., Conway E., Sweeney T. (2023). Nutritional Strategies to Mitigate Post-Weaning Challenges in Pigs: A Focus on Glucans, Vitamin D, and Selenium. Animals.

[20] Crosbie M., Zhu C., Karrow N.A., Huber L.-A. (2021). The Effects of Partially Replacing Animal Protein Sources with Full Fat Black Soldier Fly Larvae Meal (*Hermetia illucens*) in Nursery Diets on Growth Performance, Gut Morphology, and Immune Response of Pigs. Trans. Anim. Sci..

[21] Biasato I., Renna M., Gai F., Dabbou S., Meneguz M., Perona G., Martinez S., Lajusticia A.C.B., Bergagna S., Sardi L. (2019). Partially Defatted Black Soldier Fly Larva Meal Inclusion in Piglet Diets: Effects on the Growth Performance, Nutrient Digestibility, Blood Profile, Gut Morphology and Histological Features. J. Anim. Sci. Biotechnol..

[22] Spranghers T., Michiels J., Vrancx J., Ovyn A., Eeckhout M., De Clercq P., De Smet S. (2018). Gut Antimicrobial Effects and Nutritional Value of Black Soldier Fly (*Hermetia illucens* L.) Prepupae for Weaned Piglets. Anim. Feed. Sci. Technol..

[23] Luttenschlager H., Carpentier J., Beckers Y., Wavreille J., Blecker C., Purcaro G., Maesen P., Francis F., Deville N., Finet S. (2025). Impact of a Microbial and Physical Predigestion of Food Waste on the Black Soldier Fly *Hermetia illucens* (Linnaeus, 1758) Larvae Growth and Nutritional Composition. Waste Biomass Valor..

[24] European Parliament and Council of the European Union (2011). Commission Regulation (EU) No 142/2011 of 25 February 2011 Implementing Regulation (EC) No 1069/2009 of the European Parliament and of the Council Laying down Health Rules as Regards Animal by-Products and Derived Products Not Intended for Human Consumption and Implementing Council Directive 97/78/EC as Regards Certain Samples and Items Exempt from Veterinary Checks at the Border under That Directive Text with EEA Relevance.

[25] European Parliament and Council of the European Union (2009). Regulation (EC) No 1069/2009 of the European Parliament and of the Council of 21 October 2009 Laying down Health Rules as Regards Animal by-Products and Derived Products Not Intended for Human Consumption and Repealing Regulation (EC) No 1774/2002 (Animal by-Products Regulation).

[26] Crosbie M., Zhu C., Shoveller A.K., Huber L.-A. (2020). Standardized Ileal Digestible Amino Acids and Net Energy Contents in Full Fat and Defatted Black Soldier Fly Larvae Meals (*Hermetia illucens*) Fed to Growing Pigs. Trans. Anim. Sci..

[27] Bradstreet R.B. (1954). Kjeldahl Method for Organic Nitrogen. Anal. Chem..

[28] Janssen R.H., Vincken J.-P., van den Broek L.A.M., Fogliano V., Lakemond C.M.M. (2017). Nitrogen-to-Protein Conversion Factors for Three Edible Insects: Tenebrio Molitor, Alphitobius Diaperinus, and *Hermetia illucens*. J. Agric. Food Chem..

[29] Zie M., Jacquet N., Karamoko G., Alabi T., Richel A., Karoui R., Blecker C. (2025). Characterization of a Novel Natural Protein-Polysaccharide Complex from Cashew Apple Bagasse and Its Functional Implications. Food Chem..

[30] Hirs C.H.W. (1967). [19] Performic Acid Oxidation. Methods in Enzymology.

[31] Hugli T.E., Moore S. (1972). Determination of the Tryptophan Content of Proteins by Ion Exchange Chromatography of Alkaline Hydrolysates. J. Biol. Chem..

[32] Poelaert C., Francis F., Alabi T., Megido R.C., Crahay B., Bindelle J., Beckers Y. (2018). Protein Value of Two Insects, Subjected to Various Heat Treatments, Using Growing Rats and the Protein Digestibility-Corrected Amino Acid Score. J. Insects Food Feed..

[33] Hoc B., Tomson T., Malumba P., Blecker C., Jijakli M.H., Purcaro G., Francis F., Caparros Megido R. (2021). Production of Rainbow Trout (*Oncorhynchus Mykiss*) Using Black Soldier Fly (*Hermetia illucens*) Prepupae-Based Formulations with Differentiated Fatty Acid Profiles. Sci. Total Environ..

[34] Martin C., Maesen P., Minchilli D., Francis F., Verheggen F. (2021). Forensic Taphonomy: Characterization of the Gravesoil Chemistry Using a Multivariate Approach Combining Chemical and Volatile Analyses. Forensic Sci. Int..

[35] AOAC (1990). Official Methods of Analysis. Association of Official Analytical Chemists.

[36] Pinheiro J., Bates D., DebRoy S., Sarkar D., Heisterkamp S., Van Willigen B., Maintainer R. (2017). Package ‘Nlme’. Linear and Nonlinear Mixed Effects Models.

[37] Lenth R.V., Piaskowski J., Banfai B., Bolker B., Buerkner P., Giné-Vázquez I., Hervé M., Jung M., Love J., Miguez F. (2025). Emmeans: Estimated Marginal Means, Aka Least-Squares Means. Am. Stat..

[38] Zhao J., Sato M., Takao N., Ban T., Tamamaki K., Kagami M., Yano K., Kawasaki K. (2024). Defatted Black Soldier Fly Larvae Meal in Lactating Sow and Pre-Weaning Piglet Diets: Impacts on Growth Performance, Fecal Microbiota, and Metabolic Pathways. J. Insects Food Feed.

[39] Boontiam W., Phaengphairee P., Hong J., Kim Y.Y. (2022). Full-Fatted *Hermetia illucens* Larva as a Protein Alternative: Effects on Weaning Pig Growth Performance, Gut Health, and Antioxidant Status under Poor Sanitary Conditions. J. Appl. Anim. Res..

[40] Håkenåsen I.M., Grepperud G.H., Hansen J.Ø., Øverland M., Ånestad R.M., Mydland L.T. (2021). Full-Fat Insect Meal in Pelleted Diets for Weaned Piglets: Effects on Growth Performance, Nutrient Digestibility, Gastrointestinal Function, and Microbiota. Anim. Feed. Sci. Technol..

[41] Malla N., Roos N., Van der Heide M.E., Nørgaard J.V. (2024). Effect of Feeding Meal of Yellow and Lesser Mealworm and Defatted Black Soldier Fly Larvae on Growth Performance and Gut Health of Weaned Piglets. Anim. Feed. Sci. Technol..

[42] Rezaei R., Wang W., Wu Z., Dai Z., Wang J., Wu G. (2013). Biochemical and Physiological Bases for Utilization of Dietary Amino Acids by Young Pigs. J. Anim. Sci. Biotechnol..

[43] Humphrey D.C., Haydon K., Greiner L.L. (2023). Evaluation of Branched-Chain Amino Acid Interactions in 10 to 20 Kg Nursery Pigs Using a Central Composite Design. J. Anim. Sci..

[44] Siebert D., Khan D.R., Torrallardona D., Siebert D., Khan D.R., Torrallardona D. (2021). The Optimal Valine to Lysine Ratio for Performance Parameters in Weaned Piglets. Animals.

[45] Wu G. (2014). Dietary Requirements of Synthesizable Amino Acids by Animals: A Paradigm Shift in Protein Nutrition. J. Anim. Sci. Biotechnol..

[46] Yu M., Li Z., Chen W., Rong T., Wang G., Li J., Ma X. (2019). Use of *Hermetia illucens* Larvae as a Dietary Protein Source: Effects on Growth Performance, Carcass Traits, and Meat Quality in Finishing Pigs. Meat Sci..

[47] Zhu M., Liu M., Yuan B., Jin X., Zhang X., Xie G., Wang Z., Lv Y., Wang W., Huang Y. (2022). Growth Performance and Meat Quality of Growing Pigs Fed with Black Soldier Fly (*Hermetia illucens*) Larvae as Alternative Protein Source. Processes.

[48] Pânzaru R.L., Firoiu D., Ionescu G.H., Ciobanu A., Medelete D.M., Pîrvu R., Pânzaru R.L., Firoiu D., Ionescu G.H., Ciobanu A. (2023). Organic Agriculture in the Context of 2030 Agenda Implementation in European Union Countries. Sustainability.

[49] Cho I., Kong C. (2025). Growth Performance of Pigs Fed Low-Protein Diets Supplemented with Crystalline Amino Acids at Different Growth Stages. Anim. Biosci..

[50] Duarte M.E., Parnsen W., Zhang S., Abreu M.L.T., Kim S.W. (2024). Low Crude Protein Formulation with Supplemental Amino Acids for Its Impacts on Intestinal Health and Growth Performance of Growing-Finishing Pigs. J. Anim. Sci. Biotechnol..

[51] Akowuah C.F., Pan Y., Shi Z., Liu X., He R., Lü P. (2025). Revolutionizing Aquaculture Feeds: Insights into Black Soldier Fly Utilization. Aquac. Eng..

[52] El-Hack M.E.A., Shafi M.E., Alghamdi W.Y., Abdelnour S.A., Shehata A.M., Noreldin A.E., Ashour E.A., Swelum A.A., Al-Sagan A.A., Alkhateeb M. (2020). Black Soldier Fly (*Hermetia illucens*) Meal as a Promising Feed Ingredient for Poultry: A Comprehensive Review. Agriculture.

[53] Purkayastha D., Sarkar S. (2022). Black Soldier Fly Larvae for Treatment and Segregation of Commingled Municipal Solid Waste at Different Environmental Conditions. J. Environ. Manag..

[54] Shumo M., Osuga I.M., Khamis F.M., Tanga C.M., Fiaboe K.K.M., Subramanian S., Ekesi S., van Huis A., Borgemeister C. (2019). The Nutritive Value of Black Soldier Fly Larvae Reared on Common Organic Waste Streams in Kenya. Sci. Rep..

[55] Smets R., Verbinnen B., Van De Voorde I., Aerts G., Claes J., Van Der Borght M. (2020). Sequential Extraction and Characterisation of Lipids, Proteins, and Chitin from Black Soldier Fly (*Hermetia illucens*) Larvae, Prepupae, and Pupae. Waste Biomass Valor..

[56] Schiavone A., De Marco M., Martínez S., Dabbou S., Renna M., Madrid J., Hernandez F., Rotolo L., Costa P., Gai F. (2017). Nutritional Value of a Partially Defatted and a Highly Defatted Black Soldier Fly Larvae (*Hermetia illucens* L.) Meal for Broiler Chickens: Apparent Nutrient Digestibility, Apparent Metabolizable Energy and Apparent Ileal Amino Acid Digestibility. J. Anim. Sci. Biotechnol..

[57] Prachumchai R., Cherdthong A., Prachumchai R., Cherdthong A. (2023). Black Soldier Fly Larva Oil in Diets with Roughage to Concentrate Ratios on Fermentation Characteristics, Degradability, and Methane Generation. Animals.

[58] Prachumchai R., Suntara C., Kanakai N., Cherdthong A. (2025). Inclusion of Black Soldier Fly Larval Oil in Ruminant Diets Influences Feed Consumption, Nutritional Digestibility, Ruminal Characteristics, and Methane Estimation in Thai-Indigenous Steers. J. Anim. Physiol. Anim. Nutr..

[59] Xiong J., Mao J., Wang T., Feng W., Wang W., Yang C., Miao X., Wang C. (2020). Refining and Sulfurization of Oil from Black Soldier Fly and Its Application as Biodegradable Lubricant Additive. J. Am. Oil Chem. Soc..

[60] Leong S.Y., Kutty S.R.M., Malakahmad A., Tan C.K. (2016). Feasibility Study of Biodiesel Production Using Lipids of *Hermetia illucens* Larva Fed with Organic Waste. Waste Manag..

[61] Nguyen H.C., Liang S.-H., Li S.-Y., Su C.-H., Chien C.-C., Chen Y.-J., Huong D.T.M. (2018). Direct Transesterification of Black Soldier Fly Larvae (*Hermetia illucens*) for Biodiesel Production. J. Taiwan Inst. Chem. Eng..

[62] Abd Manan F., Yeoh Y.-K., Chai T.-T., Wong F.-C. (2024). Unlocking the Potential of Black Soldier Fly Frass as a Sustainable Organic Fertilizer: A Review of Recent Studies. J. Environ. Manag..

[63] Spranghers T. (2025). The Belgian Paradox: Leading in Insect Research, Lagging in Production. J. Insects Food Feed..

[64] Veldkamp T., Meijer N., Alleweldt F., Deruytter D., Campenhout L.V., Gasco L., Roos N., Smetana S., Fernandes A., van der Fels-Klerx H.J. (2022). Overcoming Technical and Market Barriers to Enable Sustainable Large-Scale Production and Consumption of Insect Proteins in Europe: A SUSINCHAIN Perspective. Insects.

[65] Bosch G., van Zanten H.H.E., Zamprogna A., Veenenbos M., Meijer N.P., van der Fels-Klerx H.J., van Loon J.J.A. (2019). Conversion of Organic Resources by Black Soldier Fly Larvae: Legislation, Efficiency and Environmental Impact. J. Clean. Prod..

[66] Meijer N., Safitri R.A., Tao W., Hoek-Van den Hil E.F. (2025). Review: European Union Legislation and Regulatory Framework for Edible Insect Production—Safety Issues. Animal.

